# γ‐T3 Increases the Sensitization of Gastric Cancer Cells to OXA by a Regulation of ARHGAP29

**DOI:** 10.1002/jbt.71119

**Published:** 2026-10-30

**Authors:** Hao Zhu, Fa‐Lin Wang, Shuang Zhang, Ting‐Ting Yang, Guang‐Qiang Gao, Hong Tian, Li Xue, Hong‐Wei Dong, Qi Wang, Shu‐Jun Zhang, Ming Liu, Jia‐Ren Liu

**Affiliations:** ^1^ Department of Clinical Laboratory The Forth Affiliated Hospital of Harbin Medical University Harbin China; ^2^ Department of Endoncrology The Second Affiliated Hospital of Harbin Medical University Harbin China; ^3^ Department of Cardiovascular Ultrasound The Forth Affiliated Hospital of Harbin Medical University Harbin China; ^4^ College of Public Health Harbin Medical University Harbin China; ^5^ Department of Pathology The Forth Affiliated Hospital of Harbin Medical University Harbin China; ^6^ Department of General Surgery The Forth Affiliated Hospital of Harbin Medical University Harbin China

**Keywords:** ARHGAP29 gene, autophagy, gastric cancer, oxaliplatin, γ‐T3

## Abstract

γ‐Tocotrienol (γ‐T3) exhibits potent anti‐cancer effects in vitro and in vivo. Our previous study demonstrated that γ‐T3 modulates autophagy activity in gastric cancer (GC) cells via the GSK‐3β/β‐Catenin pathway. In this study, we further explored whether γ‐T3 also enhances the chemosensitivity of GC cells to oxaliplatin (OXA) by regulating ARHGAP29, and verified the direct binding between γ‐T3 and ARHGAP29 as well as the physical interaction of ARHGAP29 with GSK‐3β. We employed cell viability assays, flow cytometry, tumor cell spheroid formation assays, RT‐qPCR, Western blot analysis, and stable overexpression transfection. Additionally, cellular thermal shift assay (CETSA), co‐immunoprecipitation (Co‐IP), and chloroquine (CQ) rescue CCK‐8 assays were performed for molecular interaction and autophagy function verification; we established a xenografted tumor model using MKN45 cells in BALB/c nude mice (nu/nu). γ‐T3 and OXA inhibited GC cell proliferation in vitro and in vivo, with a synergistic effect when combined (CI = 0.55 in MKN45 cells). In a mouse model, intraperitoneal injection of γ‐T3 (20 mg/kg b.w.) and OXA (2.0 mg/kg b.w.) suppressed MKN45 cell xenograft growth. This was related to cell cycle arrest and inhibition of cancer stem cells (CSCs). γ‐T3 alone or combined with OXA altered autophagy‐related protein levels after 48 h treatment, with elevated LC3‐II/LC3‐I ratio and Beclin1 expression, and reduced p62 abundance, functional rescue with autophagy inhibitor CQ confirmed that the changed autophagy contributes to drug‐induced cell death, and this phenotypic change was accompanied by suppressed GSK‐3β/β‐Catenin pathway activity. ARHGAP29 was inversely correlated with GC prognosis; CETSA results validated the direct intracellular binding between γ‐T3 and ARHGAP29, and Co‐IP assays confirmed physical interaction between ARHGAP29 and GSK‐3β. γ‐T3 inhibited the expression of ARHGAP29 at protein and mRNA levels in a time‐ and dose‐dependent manner, whether alone or with OXA. Overexpression of ARHGAP29 reversed the synergistic effects of γ‐T3 and OXA in GC cells, further supporting the regulatory linkage between ARHGAP29 and the GSK‐3β/β‐Catenin axis. The combination of γ‐T3 and OXA represents a promising therapeutic strategy to elevate oxaliplatin sensitivity and alter autophagy‐related marker expression in GC, at least partially via the ARHGAP29‐associated modulation of GSK‐3β/β‐Catenin signaling.

## Graphical abstract

γ‐T3 combined with oxaliplatin (OXA) inhibited the proliferation of gastric cancer cells by induction autophagy via ARHGAP29/GSK‐3β/β‐Catenin pathways. γ‐T3 combined with low‐dose of OXA could exert synergistic effects on induction autophagy‐related gastric cancer cell death, which is partially mediated by down‐regulation of the ARHGAP29/GSK‐3β/β‐Catenin pathways. The evidence was expressed as the LC3‐II/LC3‐I ratio and Beclin1 expression increased, and the p62 expression decreased. Over‐expressed ARHGAP29 reversed the up‐regulation of autophagy and the inhibitory effect on gastric cancer cell proliferation.

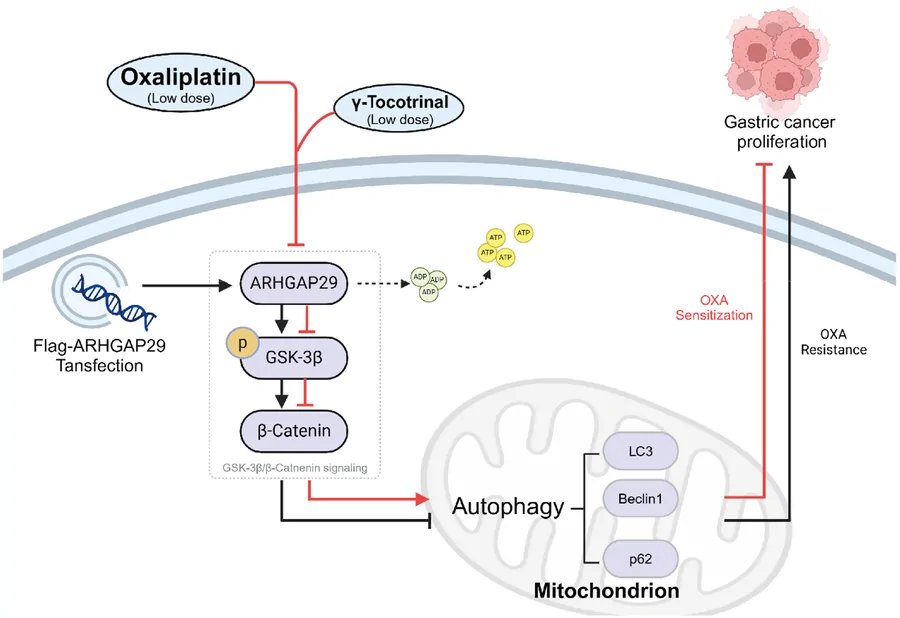

**Abbreviations**
CAP: Capecitabine
CETSA: Cellular Thermal Shift Assay
CI: Combination index
Co‐IP: Co‐Immunoprecipitation
CQ: Chloroquine
DOC: Docetaxel
EC_50_
: Median effective concentration
GC: Gastric cancer
GSK‐3β: Glycogen synthase kinase 3β
LC3: Light chain 3
OD: Optical Density
OXA: Oxaliplatin
P62: SQSTM1/p62
PCNA: Proliferating Cell Nuclear Antigen
pGSK‐3β: Phosphorylation‐glycogen synthase kinase 3β
RAGE: Receptor for advanced glycosylation end‐products
γ‐T3: γ‐Tocotrienol

## 1 Introduction

Gastric cancer (GC) causes the third highest mortality among all malignancies worldwide, and it is especially prevalent in Asia. Most patients are diagnosed with GC at the advanced stage, and the overall survival rate is less than 50% [1]. For advanced GC, long‐term administration of chemotherapeutic drugs such as oxaliplatin (OXA) frequently results in severe chemoresistance and adverse side effects [2]. Therefore, exploring feasible strategies to improve tumor chemosensitivity and identifying novel therapeutic targets may provide valuable clues for clinical GC treatment.

Phytochemicals refer to primary or secondary metabolites produced by plant metabolism. A large number of phytochemicals have been validated to exert tumor chemopreventive effects, among which curcumin [3], resveratrol [4], and quercetin [5] are capable of sensitizing tumor cells to chemotherapeutics. Among these natural compounds, γ‐Tocotrienol (γ‐T3), a subclass of vitamin E, possesses potent anti‐inflammatory, immunomodulatory and anti‐tumor capacities [6], and is abundant in oil palm, barley, walnuts and other crops [7, 8]. Previous studies have revealed that γ‐T3 elevates Adriamycin accumulation in drug‐resistant breast cancer MCF‐7/Adr cells [9], and reverses docetaxel resistance in prostate cancer PC‐3 cells [10]. Oxaliplatin (OXA), a first‐line chemotherapeutic agent recommended by CSCO and NCCN guidelines, is widely applied for GC and colorectal cancer (CRC) treatment. It suppresses DNA transcription and replication by inducing intracellular DNA crosslinks. Widespread chemoresistance limits its clinical efficacy, so OXA is often administered combined with other chemotherapeutic drugs. Our previous work has confirmed that γ‐T3 alone exerts prominent anti‐proliferative effects on GC cells [11]. Herein, we further investigated whether γ‐T3 combined with OXA could boost the chemosensitivity of GC cells, and systematically dissected the underlying molecular mechanisms of their synergistic anti‐tumor activity.

Autophagy is an evolutionarily conserved pathway maintaining cellular homeostasis, also termed type‐II programmed cell death [12]. It can exert cytoprotective effects upon cellular stress, while also restraining tumor progression via clearance of damaged organelles and oncogenic proteins [13]. Multiple bioactive phytochemicals are tightly associated with autophagy regulation; Abrus agglutinin, concanavalin A and quercetin exert anti‐tumor functions by modulating autophagy‐associated signaling cascades [14, 15, 16]. Existing studies have reported that γ‐T3 can alter autophagy activity in various tumor cell models [17, 18, 19]. Our prior data further indicated that γ‐T3 modulates autophagy and inhibits MKN45 cell xenograft growth by repressing GSK‐3β/β‐Catenin signaling [11]. Under physiological conditions, the GSK‐3β pathway serves as a negative regulator of autophagy and participates in multiple cellular biological processes. Nevertheless, extracellular stimuli can activate upstream kinases to trigger Ser9 phosphorylation and subsequent functional inactivation of GSK‐3β, which disrupts its downstream regulatory functions. Moreover, GSK‐3β and its downstream effectors regulate tumor proliferation, differentiation and apoptosis, and profoundly determine the chemotherapeutic response of malignant tumors [20]. In the current work, we sought to characterize the changes in protein levels related to autophagy triggered by γ‐T3 plus OXA co‐treatment, and identify the potential upstream regulator of the GSK‐3β/β‐Catenin axis, which constitutes the core research objective of this study.

ARHGAP29, also named PARG1 (PTPL‐associated Rho GTPase‐activating protein 1) [21], modulates Rho GTPase signaling via interacting downstream effectors as a Rho GTPase‐activating protein [22]. ARHGAP29 facilitates tumor progression by reshaping cellular energy metabolism, and its upregulation correlates positively with circulating tumor cells, metastatic glioma cells and highly invasive cancer phenotypes [23, 24]. Furthermore, ARHGAP29 has been recognized as a novel prognostic biomarker for proliferation and invasion of prostate cancer [25]. Notably, γ‐T3 also markedly suppresses ARHGAP29 expression in gastric cancer cells, which coincides with the inhibitory effect of γ‐T3 on the GSK‐3β/β‐Catenin pathway. Based on our current data of the direct binding between γ‐T3 and ARHGAP29 as well as the physical interaction between ARHGAP29 and GSK‐3β, we hypothesized that ARHGAP29 acts as an upstream regulatory factor that mediates GC tumorigenesis, progression and autophagy via modulating the activity of the GSK‐3β/β‐Catenin signaling pathway. Thus, this study aimed to explore a promising combinatorial therapeutic strategy to enhance the chemosensitivity of GC cells to OXA and alter autophagy‐related marker expression in GC cells, which is partially mediated by the regulatory interaction between ARHGAP29 and the GSK‐3β/β‐Catenin signaling pathway.

## 2 Materials and Methods

### 2.1 Cell Lines and Culture Conditions

Human gastric cancer MKN45 cells, AGS cells, and normal gastric epithelial cells (GES‐1 cells) were purchased from Procell (Wuhan, China). All cell lines were cultured in RPMI‐1640 medium (Hyclone, Beijing, China) supplemented with 10% fetal bovine serum (Procell, Wuhan, China), 1% penicillin/streptomycin (Gibco, Shanghai, China) and 1% glutamine (Beyotime, Shanghai, China). All cells were cultured in a 37°C incubator maintained at 5% CO_2_.

### 2.2 Reagents

Antibodies used in this study included LC3, Beclin1, SQSTM1/p62, phospho‐GSK‐3β (Ser9), GSK‐3β, and β‐catenin (ABclonal, Wuhan, China); β‐Actin, GAPDH, and HRP‐conjugated anti‐mouse and rabbit secondary antibodies (Cell Signaling Technology, Shanghai, China); PCNA (Beyotime, Shanghai, China); ARHGAP29 (Solarbio, Beijing, China); Rhodamine (TRITC) AffiniPure Goat anti‐Rabbit IgG and FITC‐labeled Goat Anti‐mouse IgG (ZSGB‐BIO, Beijing, China). γ‐Tocotrienol (Solarbio, Beijing, China) was dissolved in ethanol and stored at −20°C in the dark. Oxaliplatin (Sigma, Shanghai, China) was prepared as a 10‐mmol/L solution in DMSO. Chloroquine (CQ) (MedChemExpress, Shanghai, China) was prepared as a stock solution in PBS or distilled water and stored according to the manufacturer's instructions.

### 2.3 Animal Treatment

Four‐week‐old female BALB/c nude mice were purchased from the Viton Lihua Laboratory Animal Technology, Beijing, China. After 1 week adaptation, MKN45 cells (1.0 × 10^6^) were injected for initiating location at the lower part of the head at the back of the neck. The mice were randomized into the control group (30% ethanol), γ‐T3 treatment group (20 mg/kg b.w.), OXA treatment group (2.0 mg/kg b.w.), and γ‐T3 + OXA treatment group, and received intraperitoneal (IP) injections every 2 days until the nude mice were sacrificed under anesthesia (phenobarbital 50 mg/kg b.w. IP) on the 28th day. The body weight and volume of xenograft were measured every 2 days. The xenograft from each mouse was collected, measured, and weighed. The half of xenograft was fixed in 4% polyformaldehyde for immunohistochemistry or immunofluorescence. The rest of the xenograft was frozen at −80°C for subsequent Western blot. The tumor volume was calculated as: *W* ^2^ × (*L*/2), *L*: length; *W*: width. The experimental procedures were approved by the Ethics Committee of the Fourth Hospital of Harbin Medical University, China (2022‐WZYSLLSC‐12).

### 2.4 Cell Viability

Cell viability was detected by MTT and MB assays according to our previous article [26]. MKN45 cells and AGS cells were seeded in 96‐well plates and treated with various doses of γ‐T3 and/or OXA for 24, 48, or 72 h. MTT (2.0 mg/mL) or MB solution was added prior to the end of incubation. Cellular crystals were lysed with DMSO or Elusion solution, and the mixture was shaken at room temperature for 30 min. OD values were measured at 492 or 620 nm using an enzyme marker, and cell viability curves were plotted using GraphPad Prism software 5.0 (GraphPad Software LLC).

### 2.5 Docking of Molecules

Using AutoDock Vina software (http://vina.scripps.edu/), PDB database (https://www.rcsb.org/), ligand structure library (https://pubchem.ncbi.nlm.nih.gov/), pyrx software (https://pyrx.sourceforge.io/) to calculate its Affinity (kcal/mol) value which represented the binding activity. It is generally accepted that a docking energy value of less than −4.25 kcal/mol indicates some binding activity, less than −5.0 kcal/mol indicates good binding activity, and less than −7.0 kcal/mol indicates strong binding activity.

### 2.6 Cell Sphere Formation Assay

The tumorigenic properties of MKN45 cells were determined by tumor sphere formation assay and using standard techniques as described a previous study [27]. 20 ng/mL human bFGF and 50 ng/mL rhEGF were purchased from Cell Signaling Technology (CST, Shanghai, China).

### 2.7 Flow Cytometry

MKN45 cells or AGS cells (4 × 10^5^) were seeded in the bottom of a 6‐well plate overnight before being treated with γ‐T3 or/and OXA for 72 h. The cells were washed with pre‐cooled PBS, collected, and centrifuged at 2850 rpm, and fixed overnight using 75% ethanol. On the next day, repeated washing and centrifugation were followed by resuspending using 500 μL PI‐FITC (Beyotime, Shanghai, China), and stained for 30 min away from light. Finally, after centrifuged and resuspended in 500 μL PBS, the cells were analyzed by flow cytometer (BD FACSCalibur).

### 2.8 Western Blot

MKN45 cells (4 × 10^5^) were seeded in the bottom of a 6‐well plate overnight before being treated with γ‐T3 or/and OXA for different time points. The cells were washed with pre‐cooled PBS and added the lysis buffer, including RIPA (Beyotime, Shanghai, China), Complete ULTRA (Roche, Shanghai, China), and phosphatase inhibitor cocktail 2 and 3 (Sigma, Shanghai, China). Lysates were centrifuged at 14,000 rpm for 25 min and denatured at 95°C. Protein concentrations were measured using a BCA kit (Sigma‐Aldrich). Equal amounts of protein were separated by SDS‐PAGE, transferred to a PVDF membrane, and blocked with 5% nonfat milk. Membranes were incubated with primary antibodies (e.g., ARHGAP29, pGSK‐3β (Ser9), GSK‐3β, β‐Catenin, LC3, Beclin1, p62, and GAPDH) overnight at 4°C and with secondary antibodies for 2 h at room temperature. Protein bands were viewed in the ECL Chemiluminescent System (ChemiScope S6, ClinX, Shanghai, China).

### 2.9 RNA Extraction and RT‐qPCR

The total RNA from MKN45 cells or xenografts was extracted by TRIzol reagent (HaiGene, Harbin, China) and RNA extraction reagent (G‐clone, Beijing, China) according to the manufacturer's instructions. Two µg of total RNA was reverse transcribed using the PrimeScript RT reagent Kit with gDNA Eraser (Takara, Shanghai, China), and 50 ng of cDNA was used for PCR. Real‐time PCR was performed using TB Green Premix Ex Taq II (Takara, Shanghai, China) with the following conditions: 95°C for 10 min, then 40 cycles of 95°C for 15 s and 60°C for 1 min. Relative expression of the target gene was normalized against the expression of GAPDH. Relative mRNA expression was determined using the comparative CT (2^−ΔΔCt^) method.

### 2.10 Co‐IP

MKN45 cells in logarithmic growth phase were used for co‐immunoprecipitation (Co‐IP) experiment as described a previous study [28]. Briefly, after treatments, the culture medium was discarded, and the cells were washed twice with precooled PBS. RIPA lysis buffer containing protease and phosphatase inhibitors was added, and the cells were lysed thoroughly on ice for 30 min. The cell lysate was collected, centrifuged at 12,000 rpm/min for 15 min at 4°C, and the supernatant was aspirated. A small amount of the supernatant was taken as the Input sample. The remaining supernatant was divided into four aliquots, which were used for IgG negative control, ARHGAP29 immunoprecipitation, and GSK‐3β immunoprecipitation, respectively. Equal amounts of isotype IgG, anti‐ARHGAP29 antibody, and anti‐GSK‐3β antibody were added to the corresponding supernatants (total protein 1.0 mg), and the mixtures were incubated slowly overnight on a shaker at 4°C. The next day, Protein A/G agarose beads were added, and the antigen‐antibody complexes were incubated for 4 h on a shaker at 4°C. The agarose beads were collected by low‐speed centrifugation, and the beads were washed five times with precooled RIPA buffer to remove nonspecific binding proteins. 5× protein loading buffer was added to the beads and Input sample, and the mixture was boiled in a metal bath at 100°C for 10 min to fully denature. After cooling, SDS‐PAGE electrophoresis, membrane transfer, and blocking were performed. Primary antibodies against ARHGAP29 and GSK‐3β were incubated separately, and protein expression in each group was detected by Western blot.

### 2.11 CETSA

MKN45 cells were routinely seeded and cultured in two groups: the control group (treated with solvent) and the γ‐T3 group; the culture medium was discarded, the cell pellet was collected with PBS, resuspend the cells in PBS containing protease inhibitor, and aliquot into 7 tubes; Place 7 tubes in metal baths at 4°C, 50°C, 55°C, 60°C, 65°C, 70°C, and 75°C, respectively, and heat for 3 min; All samples were uniformly cooled on ice for 5 min, and the cells were lysed by repeated freeze‐thaw cycles three times; After centrifuged at 12,000 rpm/min and 4°C for 15 min, the soluble protein was carefully aspirated from the supernatant, the supernatant was added to 5× protein loading buffer and then boiled for denaturation. Western blot was performed to detect the expression of ARHGAP29 protein. After comparing the residual amount of ARHGAP29 protein in the two groups at different temperatures, the changes in protein thermal stability were analyzed.

### 2.12 IHC and Immunofluorescence

Xenograft sections were deparaffinized, and antigen retrieval was performed using 10 mmol/L sodium citrate buffer (pH = 6.0). Endogenous peroxidase was blocked with 3% H_2_O_2_ for 10 min, followed by nonspecific protein blocking. Sections were incubated with primary (1:50, PCNA) and secondary antibodies (1:50, goat anti‐mouse IgG), then stained with DAB (Beyotime) for 2–3 min and counterstained with hematoxylin. After dehydration and sealing with neutral resin, sections were observed under a microscope. The number of positive cells was counted in three fields per section at 400× magnification and averaged [29]. For immunofluorescence, primary antibodies (1:50, LC3, Beclin1, or p62) were conjugated to secondary antibodies (1:50, FITC‐goat anti‐mouse IgG or TRITC‐goat anti‐rabbit IgG). DAPI (Beyotime) was used for nuclear staining, omitting DAB and hematoxylin steps.

### 2.13 Overexpression of ARHGAP29

ARHGAP29 plasmid was purchased from VectorBuilder (Guangzhou, China). The pLV‐Puro‐CMV>Arhgap29[NM_001328664.2](ns)/FLAG:T2A:EGFP was transfected into MKN45 cells seeded in 6‐well plates when reaching 30% confluence. Lipofectamine 3000 reagent was purchased from Invitrogen (ThermoFisher SCIENTIFIC, Shanghai, China), and the entire transfection process was followed by Lipofectamine 3000 Reagent Protocol (Thermofisher.com/support). After transfected for 3 days, the infectious efficiency was evaluated by observing the FLAG expression with an inverted phase contrast microscope (Leica, Germany).

### 2.14 Statistical Analysis

All statistical analyses were performed using GraphPad InStat software (GraphPad Software Inc.). All data are presented as mean ± standard deviation (S.D.), and all experiments were repeated three independent times. For comparisons between two groups, two‐tailed unpaired Student's *t*‐test was adopted. One‐Way or Two‐Way analysis of variance (ANOVA) was used for comparisons among three or more groups, followed by Dunnett's test (comparison with a single control group) or Tukey's HSD test (all pairwise comparisons) for multiple comparison correction. *p* < 0.05 was defined as statistically significant.

## 3 Results

### 3.1 γ‐T3 and OXA Inhibited Proliferation of Gastric Cancer (GC) Cells

The MTT and MB assays showed that γ‐T3 (0–90 µmol/L) or OXA (0–5 µmol/L) treatment significantly reduced the viability of MKN45 cells and AGS cells in a time‐dose‐dependent manner (*p* < 0.01) (Figure 1A,B). The 72 h EC_50_ of MKN45 cells treated with γ‐T3 or OXA were (52.57 ± 12.57) and (1.19 ± 0.24) µmol/L, while those for AGS cells were (57.82 ± 4.52) and (3.27 ± 1.71) µmol/L, respectively (Tables 1 and 2). The above EC_50_ values were used as a basis to design the doses of combination treatment. γ‐T3 and OXA exerted synergistic anti‐proliferative effects; MKN45 cells and AGS cells were exposed to γ‐T3 combined with OXA for 72 h significantly reduced cell viability at a greater extent than that of γ‐T3 or OXA alone (*p* < 0.01) (Figure 1C,D).

**Figure 1:**
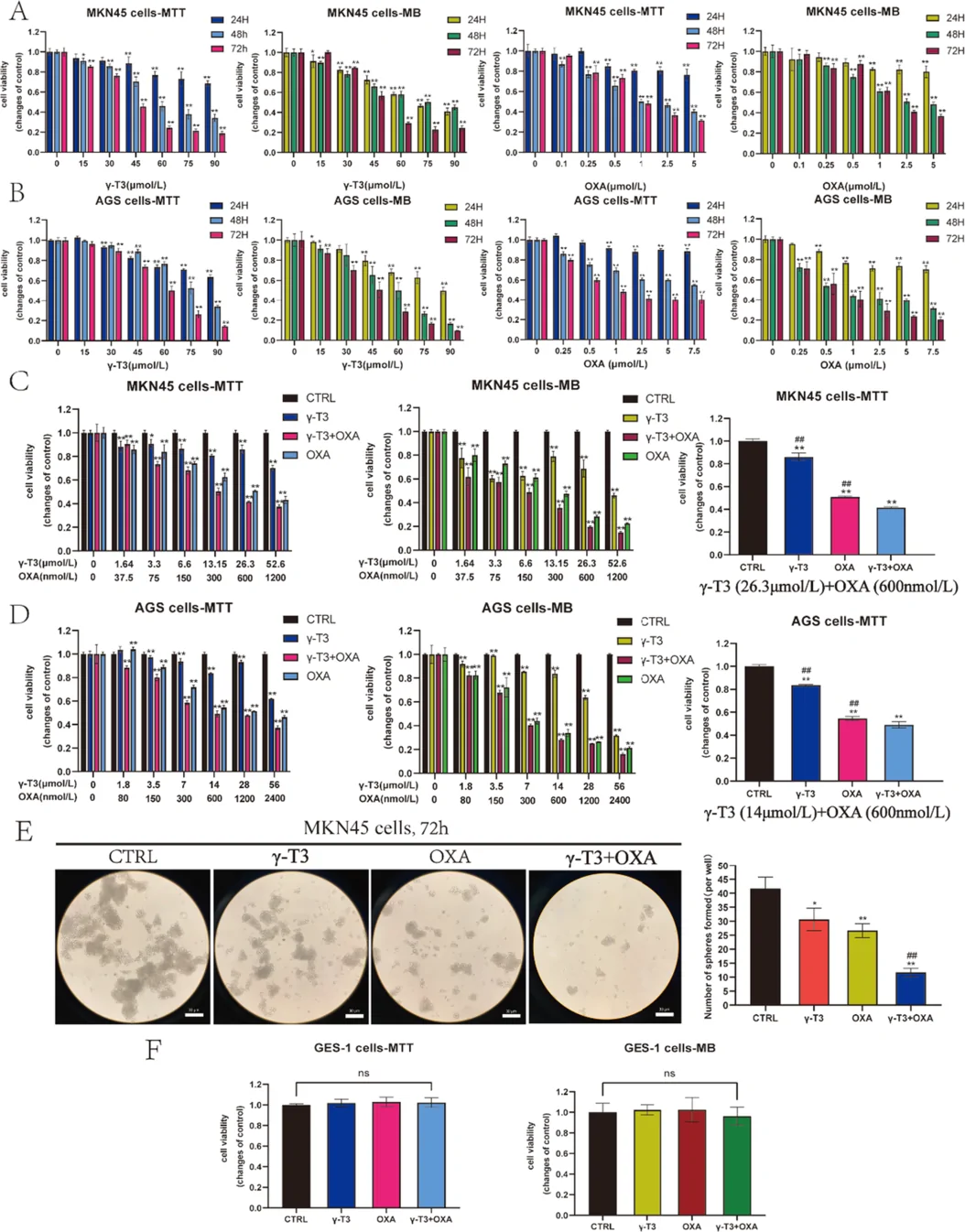
γ‐T3 and OXA inhibited the proliferation of gastric cancer cells. (A, B) MKN45 cells and AGS cells were treated with γ‐T3 or OXA for 24, 48, and 72 h; (C, D, F) MKN45 cells, AGS cells and GES‐1 cells were treated with γ‐T3 (26.3 µmol/L) and/or OXA (600 nmol/L) for 72 h. Cell viability was assessed by MTT and MB assays; (E) Cell spheroid formation assay in MKN45 cells. Data are presented as mean ± S.D. from three independent biological replicates. *p < 0.05, **p < 0.01 vs. control. Statistical analysis: Two‐tailed unpaired Student's *t*‐test for two‐group comparisons; One‐way ANOVA followed by Dunnett's test (vs. single control) or Tukey's HSD test (all pairwise comparisons).

**Table 1:** EC_50_ results of γ‐T3 or OXA treatment for 72 h in MKN45 cells.

EC_50_ (μmol/L), 72 h				MEAN ± S.D. (μmol/L)
γ‐T3	65.9218	40.9527	50.8379	52.5708 ± 12.5744
OXA	1.04561	1.46679	1.06213	1.19151 ± 0.2385

**Table 2:** EC_50_ results of γ‐T3 or OXA treatment for 72 h in AGS cells.

EC_50_ (μmol/L), 72 h				MEAN ± S.D. (μmol/L)
γ‐T3	55.5521	63.0371	54.8895	57.8262 ± 4.5248
OXA	1.8680	5.1762	2.7729	3.2723 ± 1.7097

The combination index (CI) for MKN45 cells (0.55) and AGS cells (0.59) were calculated by CompuSyn Software (Tables 3 and 4), indicated a synergistic effect of γ‐T3 and OXA on proliferation of GC cells (CI < 1). Based on these values, the doses of γ‐T3 combined with OXA were selected as 26.3 µmol/L γ‐T3 + 0.6 µmol/L OXA for MKN45 cells, and 14.0 µmol/L γ‐T3 + 0.6 µmol/L OXA for AGS cells. The cell spheroid formation assay showed that γ‐T3 or OXA alone reduced the number and size of tumor spheres, while their combination had a stronger inhibitory effect on the spheroid formation in MKN45 cells (*p* < 0.01) (Figure 1E). Importantly, after continuous treatment with 26.3 µmol/L γ‐T3 and 0.6 µmol/L OXA for 72 h, γ‐T3 combined with OXA did not significantly affect the viability of GES‐1 cells (Figure 1F).

**Table 3:** Combination index (CI) of different doses at γ‐T3 and OXA in MKN45 cells.

Total dose	Fa	CI
1.6775	0.28065	0.62617
3.37546	0.28515	1.16794
6.75091	0.30709	1.66563
13.4507	0.46288	0.62114
26.9014	0.55512	0.54596
53.8027	0.60180	0.71713

**Table 4:** Combination index (CI) of different doses at γ‐T3 and OXA in AGS cells.

Total dose	Fa	CI
1.88	0.884	0.427
3.65	0.801	0.488
7.30	0.586	0.410
14.60	0.490	0.595
29.20	0.477	1.140
58.40	0.372	1.599

### 3.2 γ‐T3 + OXA Inhibited Growth of MKN45 Cell Xenografts

To confirm the anti‐GC efficacy of γ‐T3 and OXA in a mouse model, MKN45 cell xenografts were established using BALB/c nude mice (nu/nu). The mice were randomly divided into the control group (30% ethanol), the γ‐T3 group (20 mg/kg b.w.), the OXA group (2.0 mg/kg b.w.), and the γ‐T3 + OXA group. The body weight was measured in each group, and no significant weight fluctuation was observed across all groups (Figure 2A). γ‐T3 or OXA alone inhibited xenograft growth, whereas γ‐T3 combined with OXA produced a significant synergistic suppressive efficacy (Figure 2A). The average xenograft weight and volume are shown in Figure 2B (*p* < 0.01), with the lowest tumor weight and volume detected in the γ‐T3 + OXA group. HE staining revealed that γ‐T3 or OXA alone significantly reduced tumor heterogeneity and increased lymphocyte infiltration, accompanied by increased necrotic areas relative to the control group. These phenotypes were most prominent in the γ‐T3 + OXA group (Figure 2C). The expression of PCNA (Proliferating cell nuclear antigen) was downregulated in MKN45 cell xenografts treated with γ‐T3 or OXA alone or their combination compared with the control group (*p* < 0.01) (Figure 2D).

**Figure 2:**
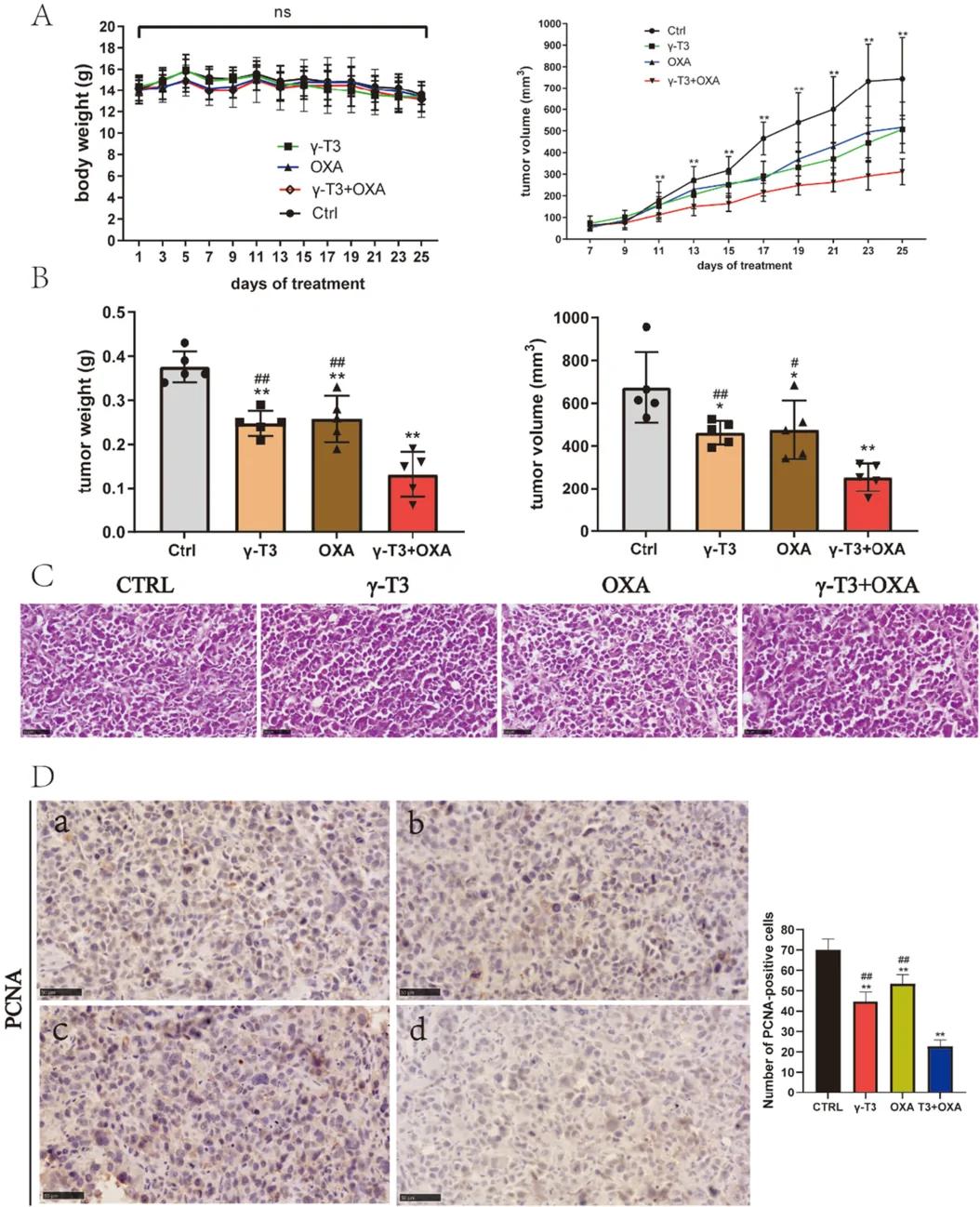
The combination of γ‐T3 and OXA inhibited the growth of MKN45 cell xenografts. (A) Effect of γ‐T3 and OXA on body weight and tumor volume in mice; (B) Average weight and volume of xenografts were suppressed by γ‐T3 and OXA; (C) HE staining of xenograft sections. Scale bar = 50 μm, 40× objective lens; (D) The positive cellular number of PCNA expression in xenograft sections (40×). Scale bar = 50 μm. a: CTRL, b: γ‐T3, c: OXA, d: γ‐T3 + OXA. *p < 0.05, **p < 0.01, when compared with the control; ^#^ *p* < 0.05, ^##^ *p* < 0.01, when compared with the γ‐T3 + OXA group. Statistical analysis: One‐way ANOVA followed by Tukey's HSD test. Data are presented as mean ± S.D. from three independent biological replicates.

### 3.3 γ‐T3 + OXA Arrested Cell Cycle Distribution in Gastric Cancer Cells

To explore why the proliferation of GC is suppressed by γ‐T3 and OXA, flow cytometry was used to detect the cell cycle distribution of the MKN45 cells and AGS cells. γ‐T3 alone arrested the MKN45 cell cycle at the G0/G1 phase, while OXA alone or combined with γ‐T3 arrested the MKN45 cell cycle at the S phase, increasing from (28.24 ± 0.01)% to (35.58 ± 0.01)% (*p* < 0.01, Figure 3A). Notably, γ‐T3 combined with OXA also arrested the AGS cell cycle at the G2/M phase (*p* < 0.01) (Figure 3B).

**Figure 3:**
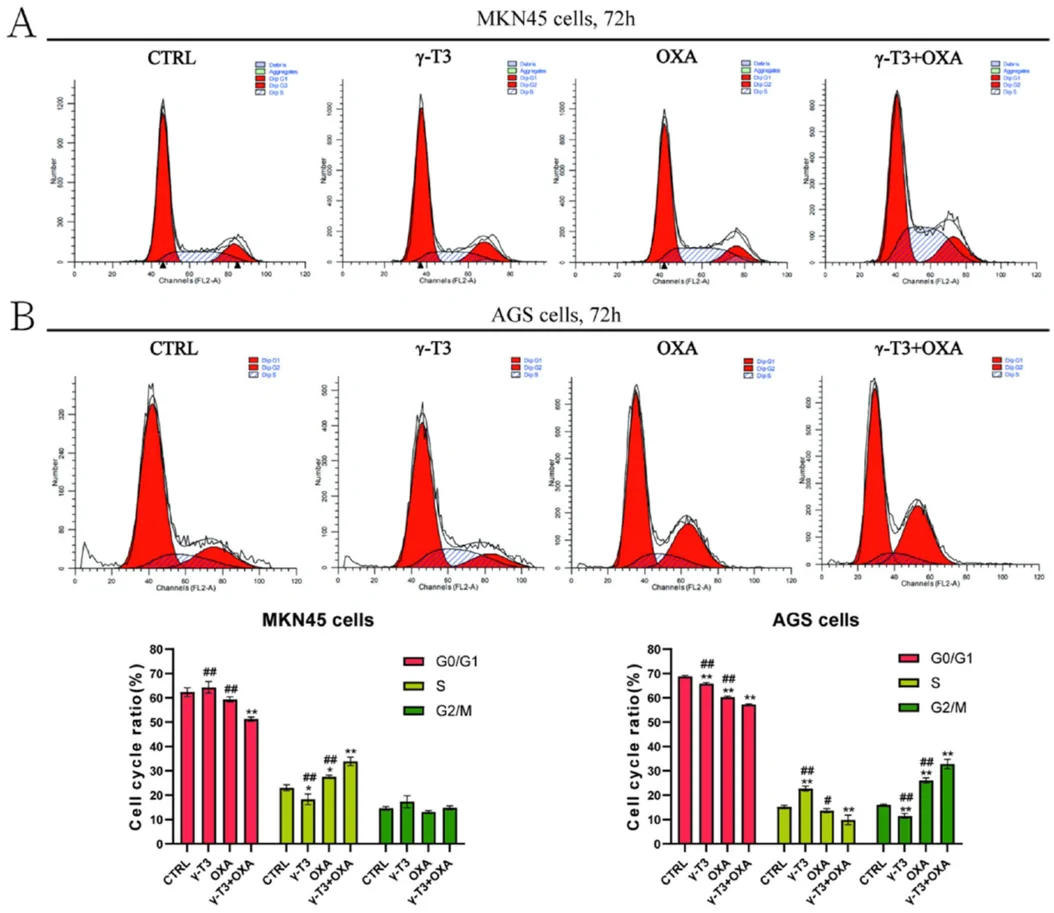
γ‐T3 and OXA arrested cell cycle in gastric cancer cells. (A) Flow cytometry was used to detect the cell cycle distribution of MKN45 cells treated with γ‐T3 (26.3 µmol/L) and OXA (600 nmol/L) for 72 h; (B) Flow cytometry was used to detect the cycle distribution of AGS cells treated with γ‐T3 (26.3 µmol/L) and OXA (600 nmol/L) for 72 h. * *p* < 0.05, ** *p* < 0.01, when compared with the control group; ^#^ *p* < 0.05, ^##^ *p* < 0.01, when compared with the γ‐T3 + OXA group. Statistical analysis: One‐way ANOVA followed by Tukey's HSD test. Data are presented as mean ± S.D. from three independent biological replicates.

### 3.4 γ‐T3 + OXA Altered Autophagy‐Related Marker Expression in Gastric Cancer Cells

To characterize changes of autophagy‐related proteins following γ‐T3 + OXA exposure in GC cells, MKN45 cells were treated with γ‐T3 (26.3 µmol/L) and OXA (600 nmol/L) for 6, 12, 24, 48 and 72 h. The protein markers related to autophagy changed in a time‐dependent manner and showed the most pronounced changes at 48 h (*p* < 0.01) (Figure 4A). Specifically, Western blot analysis also revealed an elevated LC3‐II/LC3 ‐ I ratio, upregulated Beclin1 expression, and reduced p62 abundance at 48 h. Given the most evident changes in autophagy markers at 48 h, this time point was selected for subsequent protein detection related to autophagy. γ‐T3 combined with OXA induced more prominent changes in autophagy‐related markers than γ‐T3 or OXA alone, and consistent results were observed in MKN45 xenografts (*p* < 0.01) (Figure 4B).

**Figure 4:**
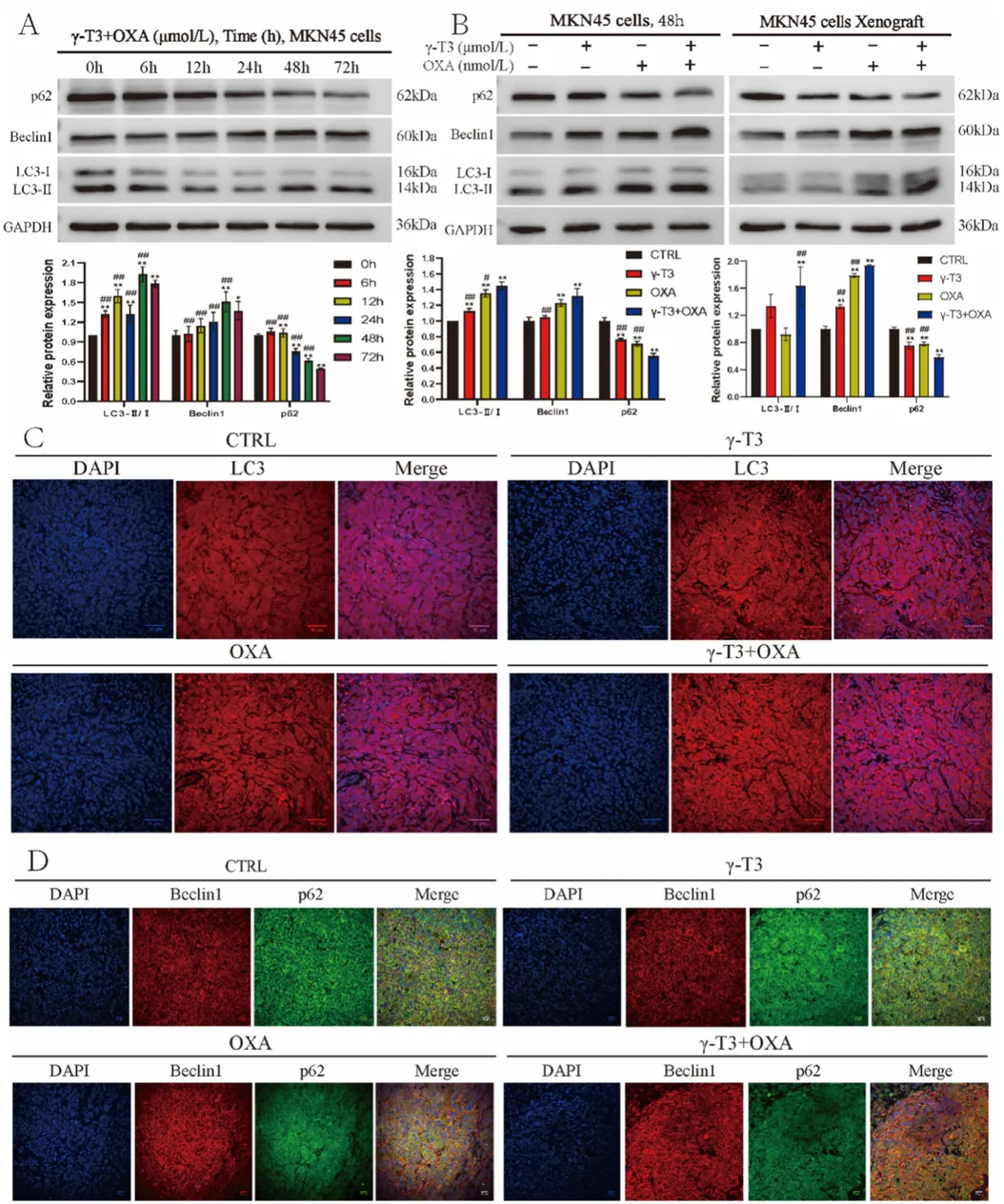
The combination of γ‐T3 and OXA altered autophagy‐related marker expression in gastric cancer cells. (A) The expression of LC3‐ Ⅱ/LC3 ‐ Ⅰ, Beclin1, and p62 proteins in MKN45 cells treated with γ‐T3 and OXA for different times; (B) The expression of LC3‐ Ⅱ/LC3‐ 1, Beclin1, and p62 proteins in xenografts treated with γ‐T3, OXA, or their combination in MKN45 cells and xenografts; (C) The changes of LC3‐I/II in xenografts by fluorescence (IF) (30×). Scale bar = 50 μm; (D) The changes of Beclin1 and p62 in xenografts by immunofluorescence (IF) (30×). Scale bar = 50 μm. * *p* < 0.05, ** *p* < 0.01, when compared with the control group, ^#^ *p* < 0.05, ^##^ *p* < 0.01, when compared with the γ‐T3 + OXA group. Statistical analysis: one‐way ANOVA followed by Tukey's HSD test. Data are presented as mean ± S.D. from three independent biological replicates.

To further clarify whether the altered autophagy exerts cytoprotective or cytotoxic effects in this cell model, CCK‐8 rescue assay was performed using chloroquine (CQ), a classic autophagy flux inhibitor. As shown in Figure S1C, CQ significantly reversed the viability loss induced by γ‐T3 combined with OXA in MKN45 cells, which confirms that the changes in autophagy triggered by their combination drofluorescence GC cell death, namely autophagy‐dependent cell death.

Additionally, proteins related to autophagy—LC3, Beclin1, and p62—were observed by immunofluorescence (IF) using laser confocal microscopy. The cytoplasmic LC3 fluorescence in the γ‐T3 + OXA group was stronger in MKN45 cell xenografts than that of γ‐T3 or OXA alone (Figure 4C). Co‐localization analyses also verified cytoplasmic binding between Beclin1 and p62 by IF (Figure 4D). After treatment with γ‐T3 + OXA, a increase in red Beclin1 fluorescence and an decrease in green p62 fluorescence were detected, accompanied by obvious orange‐red co‐localization signals in the γ‐T3 + OXA treated xenografts.

### 3.5 γ‐T3 + OXA Inhibited ARHGAP29 Expression and Suppressed GSK‐3β/β‐Catenin Signaling Activity in Gastriuc Cancer Cells

To investigate ARHGAP29 in GC prognosis, the latest gastric cancer dataset (STAD) from The Cancer Genome Atlas (TCGA) database was analyzed. Pan‐cancer analysis showed ARHGAP29 upregulation in various tumors, with significantly higher expression in GC tissues than precancerous tissues (Figure 5A). Kaplan–Meier survival analysis also showed that high expression of ARHGAP29 correlated with poorer overall survival in GC patients. ARHGAP29 expression was significantly higher in tumor tissues than that in adjacent noncancerous tissues, positively associated with GC clinical stage progression (Figure 5B). Molecular docking predicted strong binding affinity between ARHGAP29 and γ‐T3, with a docking energy of −7.5 kcal/mol (Figure 5C).

**Figure 5:**
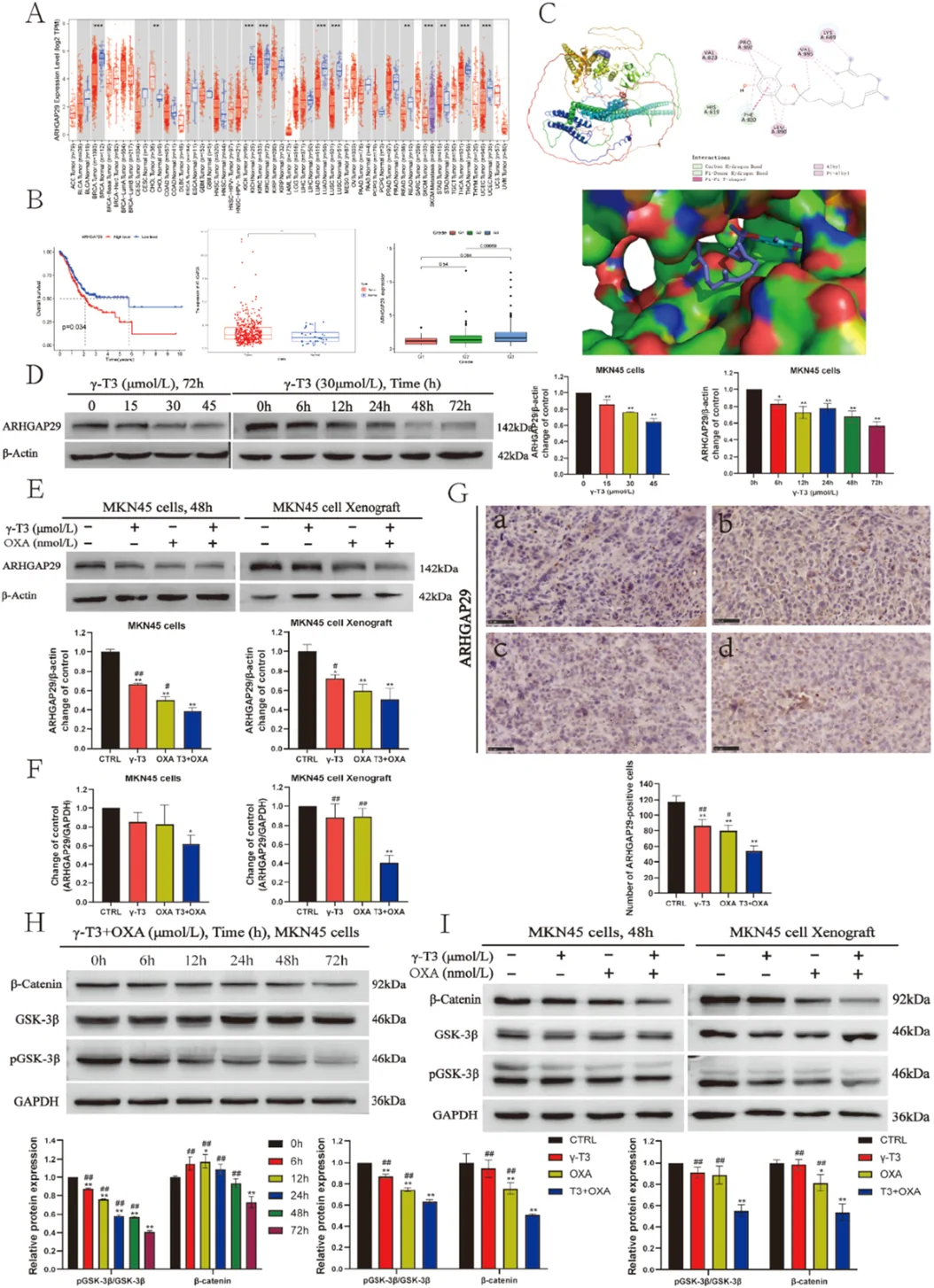
The combination of γ‐T3 with OXA inhibited ARHGAP29 and GSK‐3β/β‐Catenin signaling in gastric cancer cells. (A) ARHGAP29 expression in pan‐cancer and gastric cancer (GC) tissues (TCGA); (B) Kaplan‐Meier survival and clinical stage correlation analyses of ARHGAP29 in GC patients; (C) Molecular docking showing binding between ARHGAP29 and γ‐T3; (D) ARHGAP29 expression in MKN45 cells treated with γ‐T3 (dose‐ and time‐dependent); (E, F) The expression of ARHGAP29 at protein and mRNA levels in MKN45 cells and xenografts after γ‐T3 or OXA or their combination treatments; (G) Immunohistochemical staining of ARHGAP29 in xenografts (40×, scale bar = 50 μm); (H, I) Expression of p‐GSK‐3β (Ser9) and β‐catenin in MKN45 cells (time‐course) and xenografts after γ‐T3 or OXA or their combination treatments. Data are presented as mean ± S.D. from three independent biological replicates. *p < 0.05, **p < 0.01 vs. control; ^#^ *p* < 0.05, ^##^ *p* < 0.01 vs. γ‐T3 + OXA. Statistical analysis: One‐way ANOVA followed by Tukey's HSD test.

The cellular thermal shift assay (CETSA) was further performed to experimentally validate the predicted direct binding between γ‐T3 and ARHGAP29. MKN45 cells were divided into the solvent control group and γ‐T3 treatment group, and intact cells were heated at seven temperature points (4°C, 50°C, 55°C, 60°C, 65°C, 70°C, and 75°C). As illustrated in Figure S1C, γ‐T3 treatment markedly elevated the thermal stability of ARHGAP29 at multiple temperature points, providing experimental evidence to support the molecular docking prediction of direct small molecule‐protein binding.

Based on previous studies showing γ‐T3's inhibitory effects on GC. In the present study, we found that γ‐T3 treatment for 72 h inhibited ARHGAP29 expression in a concentration‐dependent manner. Moreover, γ‐T3 (26.3 µmol/L) inhibited ARHGAP29 expression in a time‐dependent manner (Figure 5D). Thus, we hypothesized that ARHGAP29 acts as a potential functional target mediating the anti‐tumor effects of γ‐T3 combined with OXA in GC. Western blot and RT‐qPCR analyses also showed that γ‐T3 + OXA significantly reduced ARHGAP29 mRNA and protein levels in MKN45 cells and xenografts (*p* < 0.01) (Figure 5E,F). Immunohistochemistry (IHC) staining also showed few ARHGAP29‐positive cells after γ‐T3 or OXA alone, with the strongest inhibitory effect observed in the γ‐T3 + OXA group (*p* < 0.01) (Figure 5G).

Reciprocal co‐immunoprecipitation (Co‐IP) assays were carried out in MKN45 cells to explore the physical interaction between ARHGAP29 and GSK‐3β. As shown in Figure S1A, endogenous ARHGAP29 could be pulled down by anti‐GSK‐3β antibody, and conversely, anti‐ARHGAP29 antibody also enriched GSK‐3β protein, which verified the intracellular physical interaction between ARHGAP29 and GSK‐3β.

We further explored the effects of γ‐T3 + OXA on GSK‐3β/β‐Catenin signaling. After treatment with γ‐T3 + OXA in MKN45 cells for 6,12, 24, 48, and 72 h, pGSK‐3β (Ser9) and β‐Catenin expression decreased dramatically in a time‐dependent manner (*p* < 0.01) (Figure 5H). Given the most evident changes in autophagy markers at 48 h, alterations of GSK‐3β/β‐Catenin signaling under the same treatment condition were detected in this study. Results showed that γ‐T3 + OXA significantly suppressed GSK‐3β/β‐Catenin signaling activity in MKN45 cells and xenografts (*p* < 0.01) (Figure 5I). Collectively, these data indicate that γ‐T3 + OXA downregulates ARHGAP29 expression and restrains the activity of the GSK‐3β/β‐Catenin signaling cascade.

### 3.6 Overexpression ARHGAP29 Attenuated the Synergistic Anti‐Proliferative Effect of γ‐T3 + OXA on Proliferation of Gastric Cancer Cells

MKN45 cells were infected with a lentivirus overexpressing Flag‐ARHGAP29, and then WT‐MKN45 cells and Flag‐ARHGAP29‐MKN45 cells were treated with γ‐T3 and OXA. MTT and MB assays revealed that Flag‐ARHGAP29‐MKN45 cells had higher viability than WT‐MKN45 cells under γ‐T3 + OXA treatment (*p* < 0.05) (Figure 6A), indicating that ARHGAP29 overexpression attenuates the enhanced anti‐proliferative effect of γ‐T3 and OXA on GC cell proliferation. Additionally, ARHGAP29 overexpression reversed the inhibitory effects of γ‐T3 + OXA on p‐GSK‐3β and β‐Catenin expression; obvious suppression of the GSK‐3β/β‐Catenin signaling pathway was absent in Flag‐ARHGAP29‐overexpressing cells (*p* < 0.05) (Figure 6B). Consistent results were observed for autophagy‐related protein expression (LC3, Beclin1, and p62): ARHGAP29 overexpression alone altered basal autophagy‐related marker levels, and γ‐T3 + OXA failed to trigger obvious changes in autophagy‐related protein expressionin Flag‐ARHGAP29 MKN45 cells (*p* < 0.05) (Figure 6C).

**Figure 6:**
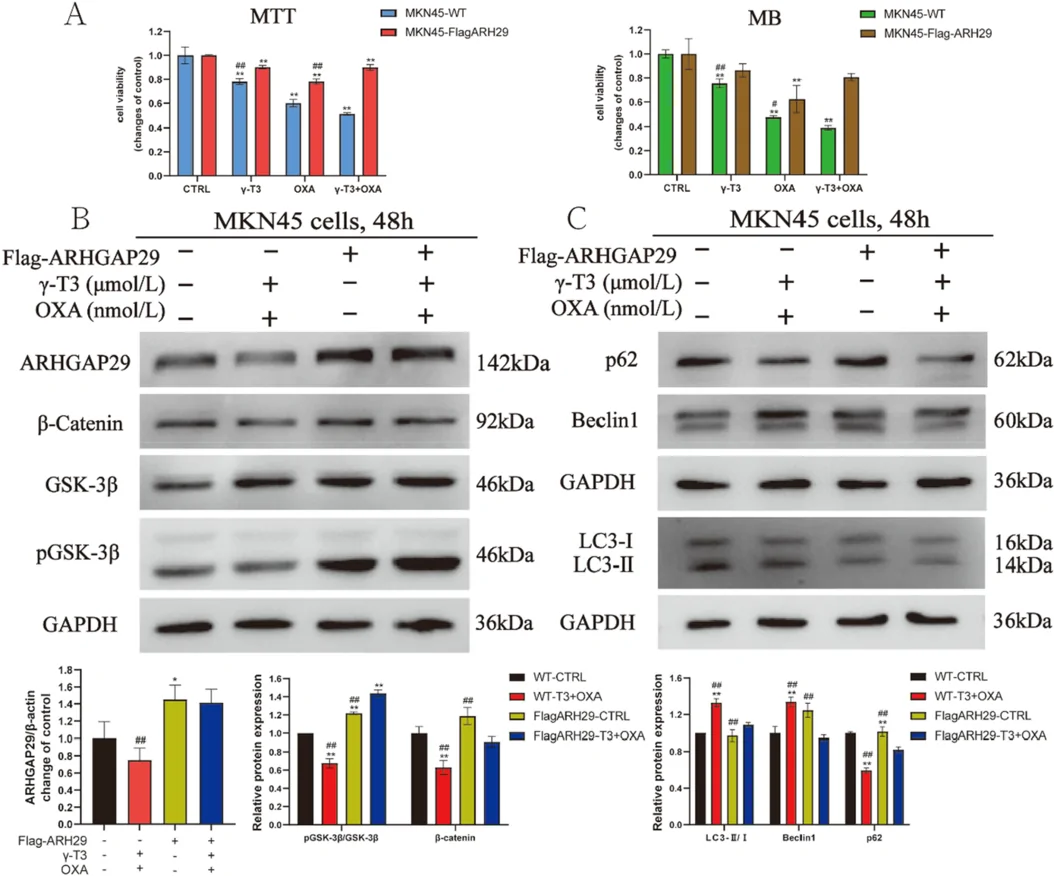
ARHGAP29 overexpression affected autophagy and chemosensitivity of gastric cancer cells via the GSK‐3β/β‐catenin pathway. (A) ARHGAP29 overexpression reduced synergistic effect of γ‐T3 combined with OXA detected by MTT and MB assays; (B) ARHGAP29 overexpression restored the GSK‐3β/β‐catenin signaling suppressed by γ‐T3 combined with OXA; (C) ARHGAP29 overexpression ARHGAP29 abrogates changes in autophagy‐related markers induced by γ‐T3 combined with OXA. * *p* < 0.05, ** *p* < 0.01, when compared with the control group; ^#^ *p* < 0.05, ^##^ *p* < 0.01, when compared with the γ‐T3 + OXA group. Statistical analysis: One‐way ANOVA followed by Tukey's HSD test. Data are presented as mean ± S.D. from three independent biological replicates.

## 4 Discussion

Combination therapy of phytochemicals and chemotherapeutic agents is effective for improving the prognosis of advanced gastric cancer (GC) patients. Preclinical studies have shown that phytochemicals, such as withaferin A combined with cisplatin, can inhibit the growth of ovarian epithelial carcinomas cells [30]. β‐Carotene enhances chemosensitivity by stimulating ATPase activity to inhibit P‐gp transcription in drug‐resistant tumor cells [6]. γ‐T3, a subtype of vitamin E, has shown promise both alone and in combination with traditional chemotherapeutic drugs. Our previous studies confirmed that γ‐T3 alone inhibits gastric cancer cell proliferation in vitro and in vivo [11, 28, 31]. Despite the promising potential of γ‐T3 for clinical translation, it still has a significant gap before being used clinically as a standalone chemotherapeutic agent like paclitaxel or vincristine. Thus, exploring the combination of γ‐T3 with OXA also reflects our expectations for its synergistic role in chemotherapy. The EC_50_ values of MKN45 cells treated with γ‐T3 or OXA alone for 72 h were (52.57 ± 12.57) µmol/L for γ‐T3 and (1.19 ± 0.24) µmol/L for OXA (Table 1). γ‐T3 (26.3 µmol/L) combined with OXA (600 nmol/L) significantly inhibited MKN45 cell viability (CI = 0.55), similar to AGS cells (CI = 0.59). MKN45 cells were more sensitive to this combination, so subsequent experiments focused on them. The MKN45 cell xenograft model showed that γ‐T3 or OXA alone inhibited xenograft growth, but their combination produced a greater inhibitory effect. Notably, unlike the previous study using a high dose of OXA (10.0 mg/kg b.w.) [32], which may cause significant toxicity, our study employed a low dose of OXA (2.0 mg/kg b.w.), which undoubtedly demonstrated the synergistic effect and safety of γ‐T3 combined with OXA.

To explore the potential mechanism of proliferation suppression in gastric cancer (GC), the effects of γ‐T3 + OXA on cancer cell stemness and gastric cancer cell cycle were examined. Cancer stem cells (CSCs), which play a key role in carcinogenesis and metastasis, are tumor cells with self‐renewal ability. A cell sphere formation assay showed that γ‐T3 + OXA significantly reduced MKN45 cell spheres. Additionally, γ‐T3 has been shown to arrest the cell cycle of GC cells at the G0/G1 phase [33, 34]. In this study, γ‐T3 alone arrested the cell cycle of MKN45 cells at the G0/G1 phase, while OXA arrested it at the S phase. Their combination also enhanced this effect, increasing the S phase from (28.24 ± 0.01)% to (35.58 ± 0.01)%. Thus, CSC inhibition and cell cycle arrest may contribute to the effects of γ‐T3 + OXA on antiproliferative capability in GC cells.

Autophagy is a complex process to degrade dysfunctional intracellular components in lysosomes [35], and phytochemicals influence this process via pathways like the PI3K/AKT/mTOR axis [14], for example, flavonoids target this axis to induce autophagy and arrest the cell cycle at the G2/M phase, leading to GC cell death [36]. Quercetin also modulates this axis to induce pancreatic cancer autophagy, reversing RAGE‐mediated gemcitabine resistance [14], but few studies have examined the correlation between autophagy and GSK‐3β/β‐Catenin signaling. Our recent research provided new evidence that γ‐T3 treatment upregulates markers related to autophagy at 30 µmol/L for 24 h via GSK‐3β/β‐Catenin signaling in MKN45 cells. In this study, MKN45 cells treated with γ‐T3 + OXA (26.3 µmol/L + 600 nmol/L) for different time points (Figure 4A) displayed obvious upregulation of protein expression related to autophagy at 48 h. Their combination treatment produced more obvious upregulation of markers related to autophagy than those of γ‐T3 or OXA alone both in MKN45 cells and MKN45 cell xenografts (Figure 4B), marked by an increased the LC3‐II/LC3 ‐Ⅰ ratio and Beclin1 expression, and decreased p62 expression. Immunofluorescence (IF) also supported our hypothesis that γ‐T3 + OXA altered the distribution of proteins related to autophagy in MKN45 cell xenografts. The fluorescent spots of LC3 were accumulated in the cytoplasm, and fluorescence co‐localization of Beclin1 and p62 showed strong binding activity. To further clarify the biological function of these altered autophagy markers, we performed chloroquine (CQ) rescue CCK‐8 assays (Figure S1C). The results suggested that the changes in autophagy‐related proteins triggered by γ‐T3 + OXA promote cell death rather than exert a cytoprotective function in GC cells. These findings above indicated that the changes in autophagy‐related markers may serve as the molecular evidence for a synergistic role of γ‐T3 + OXA in GC treatment (Figure 7).

**Figure 7:**
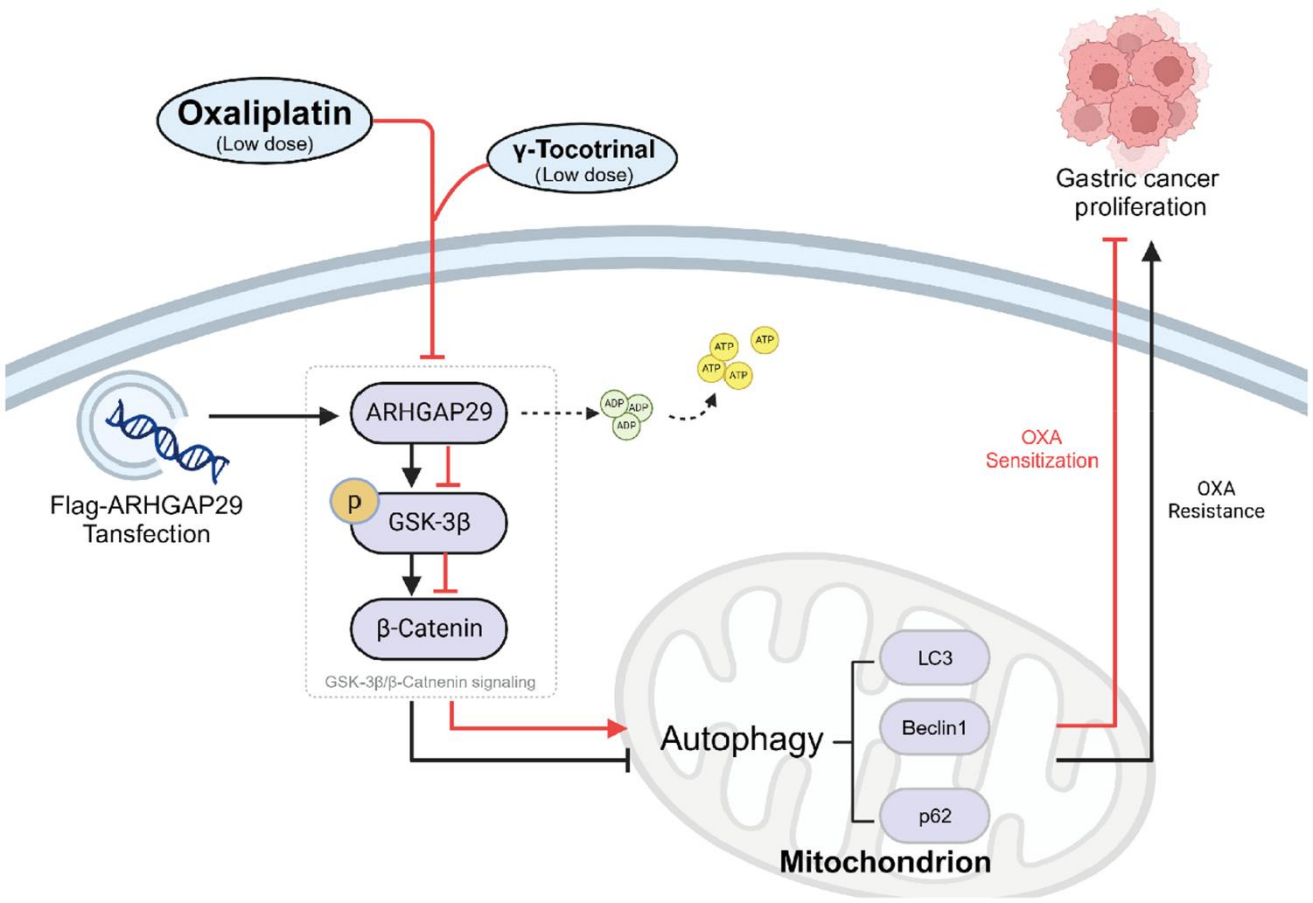
The combination of γ‐T3 and OXA alters autophagy‐related markers and suppresses gastric cancer cell proliferation via the ARHGAP29/GSK‐3β/β‐Catenin pathway. γ‐T3 could exert synergistic effects with a low‐dose of OXA to trigger autophagy‐dependent cell death in gastric cancer (GC) cells, which is partially mediated by down‐regulation of the ARHGAP29/GSK‐3β/β‐Catenin pathway. The evidence was that the LC3‐II/LC3‐I ratio and Beclin1 expression increased, and that p62 expression decreased. ARHGAP29 overexpression reversed the alterations in autophagy‐related markers and the inhibitory effect on GC cell proliferation.

ARHGAP29 gene, a small GTPase protein linked to energy metabolism, is expressed in normal tissues like the heart, skeletal muscle, and placenta [37]. It regulates breast cancer cell invasion and EMT [38] and is associated with GC cell migration via YAP signaling [24]. ARHGAP29 expression is higher in cancer tissues, negatively correlates with patient survival (Figure 5B). Molecular docking showed significant binding between ARHGAP29 and γ‐T3, and cellular thermal shift assay (CETSA) also verified this predicted binding interaction (Figure S1B). These data indicated that γ‐T3 can directly bind intracellular ARHGAP29 and elevate its thermal stability, indicating ARHGAP29 as a direct molecular target of γ‐T3. Nevertheless, techniques such as SPR or ITC can be adopted in future research to quantify detailed binding kinetic parameters, and to further determine how γ‐T3 downregulated ARHGAP29 in a time‐ and dose‐dependent manner. These results inspired us to consider that ARHGAP29 may have had some interaction with the GSK‐3β/β‐Catenin signaling pathway. Reciprocal co‐immunoprecipitation (Co‐IP) assays (Figure S1A) further verified the direct physical interaction between endogenous ARHGAP29 and GSK‐3β proteins, providing solid molecular evidence for their regulatory linkage.

In the combined therapy, the expression of ARHGAP29 at levels of mRNA and protein was decreased in MKN45 cells and xenografts by γ‐T3 + OXA, confirming our hypothesis that γ‐T3 + OXA effectively regulates ARHGAP29 expression. Additionally, their combination treatment downregulated GSK‐3β/β‐Catenin signaling, resulting in decreased pGSK‐3β (Ser9) and β‐Catenin expression. Notably, treating MKN45 cells with γ‐T3 + OXA for 48 h autophagy‐related markers, inhibited ARHGAP29 expression, and suppressed GSK‐3β/β‐Catenin signaling simultaneously. To explore the intrinsic link between ARHGAP29 and autophagy regulated by the GSK‐3β/β‐Catenin signaling pathway in GC cells, ARHGAP29‐overexpressing MKN45 cells were constructed by lentiviral‐mediated gene transfection. ARHGAP29 overexpression reversed the upregulation of autophagy‐related markers and the inhibition of GSK‐3β/β‐Catenin signaling caused by γ‐T3 + OXA, suggesting ARHGAP29 participates in modulating the activity of the GSK‐3β/β‐catenin signaling pathway. Cell viability significantly increased with ARHGAP29 overexpression in MKN45 cells, likely due to the reversal of autophagy‐dependent cell death.

Although the specific mechanisms by which ARHGAP29 activates the GSK‐3β/β‐Catenin signaling are still unclear, this is a focus for our future research. However, this study provides new evidence that γ‐T3 with low‐dose OXA can exert synergistic effects on triggering autophagy‐dependent GC cell death, partially achieved via ARHGAP29‐associated modulation of the GSK‐3β/β‐Catenin signaling pathway. ARHGAP29, acting as a potential upstream regulator of the GSK‐3β/β‐Catenin signaling pathway, participates in regulating the changes in autophagy‐related markers induced by γ‐T3 + OXA in GC cells, and offers a highly promising therapeutic target for GC treatment. In follow‐up research, shRNA‐mediated ARHGAP29 stable knockdown cell lines are planned to be constructed and to perform mRFP‐GFP‐LC3 dual fluorescence autophagy flux detection, and to carry out transcriptomic screening to identify downstream effectors of ARHGAP29, so as to further complete and refine the regulatory mechanism proposed in this work.

Several limitations exist in this work. First, autophagy flux assays with lysosomal inhibitors were not performed to distinguish whether the increased LC3‐II/LC3‐I ratio arises from enhanced autophagosome formation or impaired lysosomal clearance; Second, only ARHGAP29 overexpression cell model was used in this study, lacking reciprocal knockdown assays and corresponding detection of GSK‐3β phosphorylation and β‐catenin levels, which restricts full validation of the ARHGAP29/GSK‐3β/β‐catenin regulatory cascades; third, transcriptomic screening was not conducted to identify potential downstream mediators of ARHGAP29. Relevant knockdown validation experiments will be perfomed to improve the proposed mechanism in the future study.

## Author Contributions

**Hao Zhu:** conceptualization, data curation, formal analysis, investigation, methodology, project administration, resources, software, validation, visualization, writing – original draft. **Fa‐Lin Wang:** conceptualization, data curation, methodology, supervision, resources, writing – review and editing. **Shuang Zhang:** project administration, supervision, methodology, writing – review and editing. **Guang‐Qiang:** data curation, formal analysis, methodology, supervision. **Li Xue:** data curation, formal analysis, methodology, supervision. **Hong‐Wei Dong:** resources, visualization, writing – review and editing. **Hong Tian:** resources, visualization, writing – review and editing. **Qi Wang:** resources, visualization, writing – review and editing. **Shu‐Jun Zhang:** conceptualization, methodology, funding, writing – review and editing. **Ming Liu:** conceptualization, methodology, funding, writing – review and editing. **Jia‐Ren Liu:** conceptualization, funding acquisition, methodology, project administration, resources, supervision, writing – review and editing.

## Ethics Statement

The animal care and experimental procedures were approved by the Ethics Committee of the Fourth Hospital of Harbin Medical University, China (Approval No.: 2022‐WZYSLLSC‐12).

## Conflicts of Interest

The authors declare no conflicts of interest.

## Supporting information


Supporting File

## Data Availability

The datasets generated and/or analyzed during the current study are available from the corresponding author on reasonable request.
