# Supplementary material for: γ‐T3 Increases the Sensitization of Gastric Cancer Cells to OXA by a Regulation of ARHGAP29
Source: J Biochem Mol Toxicol. 2026 Oct 30;40(11):e71119. doi: 10.1002/jbt.71119 (PMC13720748; doi:10.1002/jbt.71119)
Supplement: Supplementary file 1 [file JBT-40-e71119-s001.docx]

**Supplementary figure S1. Supplementary validation experiments verified the interaction between γ-T3 and ARHGAP29 as well as the functional role of altered autophagy. (**A) Reciprocal co-immunoprecipitation (Co-IP) assay for endogenous ARHGAP29 and GSK-3β in MKN45 cells. Input, IgG negative control, IP-ARHGAP29 and IP-GSK-3β groups were subjected to Western blot to detect the physical binding between ARHGAP29 and GSK-3β. Three independent biological replicates were performed; **(**B) Cellular thermal shift assay (CETSA) for detecting direct binding of γ-T3 to ARHGAP29. MKN45 cell lysates with or without γ-T3 treatment were incubated at gradient temperatures (4 °C, 50 °C, 55 °C, 60 °C, 65 °C, 70 °C, and 75 °C), followed by Western blot to measure remaining soluble ARHGAP29 protein. γ-T3 treatment significantly improved the thermal stability of ARHGAP29. Three independent biological replicates were conducted; **(**C) Chloroquine (CQ) rescue CCK-8 cell viability assay in MKN45 cells. Cells were treated with γ-T3 + OXA with or without CQ co-incubation. CQ reversed the viability loss induced by γ-T3 + OXA, confirming that drug-triggered upregulated markers related to autophagy lead to autophagy-dependent cell death. Statistical analysis: One-Way ANOVA followed by Tukey’s HSD multiple comparison test. Data are presented as mean ± S.D. from three independent biological replicates. ***P* < 0.01 versus γ-T3+OXA group.
